# Multi-GNSS-Weighted Interpolated Tropospheric Delay to Improve Long-Baseline RTK Positioning

**DOI:** 10.3390/s22155570

**Published:** 2022-07-26

**Authors:** Farinaz Mirmohammadian, Jamal Asgari, Sandra Verhagen, Alireza Amiri-Simkooei

**Affiliations:** 1 Department of Geomatics Engineering, Faculty of Civil Engineering and Transportation, University of Isfahan, Isfahan 8174673441, Iran; f.mirmohammadian@tudelft.nl (F.M.); asgari@eng.ui.ac.ir (J.A.); 2 Department of Geoscience and Remote Sensing, Delft University of Technology, 2600 AA Delft, The Netherlands; a.amirisimkooei@tudelft.nl

**Keywords:** tropospheric slant delay, tropospheric zenith delay, double-difference, real-time kinematic, weighted least-squares, interpolation

## Abstract

Until now, RTK (real-time kinematic) and NRTK (Network-based RTK) have been the most popular cm-level accurate positioning approaches based on Global Navigation Satellite System (GNSS) signals in real-time. The tropospheric delay is a major source of RTK errors, especially for medium and long baselines. This source of error is difficult to quantify due to its reliance on highly variable atmospheric humidity. In this paper, we use the NRTK approach to estimate double-differenced zenith tropospheric delays alongside ambiguities and positions based on a complete set of multi-GNSS data in a sample 6-station network in Europe. The ZTD files published by IGS were used to validate the estimated ZTDs. The results confirmed a good agreement, with an average Root Mean Squares Error (RMSE) of about 12 mm. Although multiplying the unknowns makes the mathematical model less reliable in correctly fixing integer ambiguities, adding a priori interpolated ZTD as quasi-observations can improve positioning accuracy and Integer Ambiguity Resolution (IAR) performance. In this work, weighted least-squares (WLS) were performed using the interpolation of ZTD values of near reference stations of the IGS network. When using a well-known Kriging interpolation, the weights depend on the semivariogram, and a higher network density is required to obtain the correct covariance function. Hence, we used a simple interpolation strategy, which minimized the impact of altitude variability within the network. Compared to standard RTK where ZTD is assumed to be unknown, this technique improves the positioning accuracy by about 50%. It also increased the success rate for IAR by nearly 1.

## 1. Introduction

RTK positioning is the most frequently used method of achieving high accuracy with GNSS technology in (near) real-time, and network-based RTK is far more effective than single-baseline RTK in providing precise positioning over the entire area covered by reference stations [1]. The goal of relative positioning is to eliminate or reduce error sources by computing double differenced GNSS data.

Accuracy problems exist in many RTK systems due to satellite orbit errors, multipath, signal interference, ionospheric and tropospheric refraction, among others. Appropriate mitigation measures exist for some components of the RTK error budget. For instance, multi-frequency observations can be used to decrease or eliminate individual errors such as ionospheric and multipath errors [2,3]. One way to evaluate the tropospheric delay is to measure it directly with devices such as radiosondes and water vapor radiometers (WVR). However, the cost of installing these devices at each station is prohibitive. As another solution, surface pressure, temperature, and relative humidity measurements can be used in conjunction with theoretical tropospheric models such as Hopfield [4], Ifadis [5], and Mendes [6] to estimate tropospheric delays. These models often do not account for the vertical and horizontal spatial variations in atmospheric conditions, particularly the vertical profile of water vapor. Therefore, due to systematic errors caused by the atmosphere, single-base RTK performance is limited to baselines smaller than 20 km [7]. Several attempts have been made to improve RTK positioning at longer baselines by accounting for the atmospheric delay [8,9,10,11].

Compared to GPS-only RTK, the availability, reliability, positioning accuracy, and convergence speed of multi-GNSS RTK can be significantly higher. It was shown that longer baselines could be handled by increasing the number of observations together with better tropospheric models [12]. However, the tropospheric error remains one of the most critical factors in this method. For long baseline RTK, the zenith hydrostatic delay (ZHD) and the zenith wet delay (ZWD) can be corrected in advance using available models, for instance, Saastamoinen [13]. Since these models do not account for the whole wet part, the residual zenith wet delay, which is mismodeled, is usually treated as an unknown parameter in the positioning model. In this way, water vapor can also be estimated, which is known as GNSS meteorology. GNSS meteorology is getting popular and has provided promising results; the required measurements are inexpensive and available in all weather conditions [14,15]. Bevis was the first to propose this approach using GPS signals [14]. Several studies have evaluated the benefits of using a combination of constellations for tomography compared to using GPS alone [16,17]. These water vapor estimations can also improve the production of more accurate weather forecasts using Numerical Weather Prediction (NWP) models. They led to a 40% reduction in forecast errors compared to estimates from an NWP model with and without GNSS precipitable water vapor estimate assimilation [18].

An accurate estimate of the wet tropospheric delay is required to facilitate ambiguity resolution and precise positioning in the network RTK. GNSS carrier phase data can be utilized to estimate position, ambiguities, and the tropospheric zenith delay with high precision [19].

However, treating wet tropospheric delays as unknown parameters makes the mathematical model weaker and increases the observation time required to estimate the correct ambiguities. To tackle this issue, the weighted least-squares method can be used with the a priori data of the tropospheric delay of the rover station [20]. Data from a CORS GNSS network can be interpolated to forecast the tropospheric delays at any other location. These prediction data can be utilized to improve positioning accuracy, decrease the time to reach a given level of precision [21], and overcome distance restrictions [22].

Kriging interpolation has been used to compute ZTD in various studies. Kriging interpolation is a method for presenting predictions in the form of a weighted average of observations obtained at neighboring sites. The weights are chosen such that the resulting errors are minimal compared to other linear summations. Janssen confirmed that Kriging interpolation works better for tropospheric delay prediction than polynomial or spline approaches [23]. Xu et al. used Kriging interpolation to generate slant tropospheric delays in large-scale interferometric synthetic aperture radars (InSAR) to reduce tropospheric delay noise [24]. They conclude that when the spatial resolution of the reference stations is dense enough, Kriging interpolation is most efficient. Ma et al., on the other hand, show that the benefits of a dense network for the average RMSE over the year are not significant since the spatial distribution of water vapor is stable over time. Moreover, accurate predictions of the rover’s ZTD outside the network are mainly determined by the distance to a nearby reference station and are thus unaffected by the number of reference stations. In conclusion, Kriging interpolation depends on the distances between unknown points, all available measurements, and the covariance shown in the semivariogram [25].

It should be noted that, even for network RTK, tropospheric interpolation errors grow with the baseline length. Moreover, because the ZTD is correlated to the station’s height, the interpolation accuracy of this value is expected to be significantly lowered when the altitude difference between the rover and the reference stations is considerable. One strategy is to weigh tropospheric delay corrections rather than accounting for them deterministically and consider them as weighted a priori data in WLS. An extended stochastic model of GNSS measurements can be used to consider these tropospheric weights. Therefore, one of the objectives of this research is to evaluate the performance of multi-GNSS long baseline RTK with WLS using weighted a priori tropospheric data.

In this work, we use tropospheric delay modeling to derive more accurate positions for rover stations at longer inter-station distances than is currently possible. We shall use multi-GNSS code and carrier phase observations from GPS, GLONASS, Galileo, and BeiDou systems, imitating real-time rover positioning. Section 2 presents a typical DD observation model for RTK positioning with a long baseline, followed by a functional and stochastic model. Section 3 delves into the tropospheric delay in detail. Section 4 discusses the least square models for both ZTD-float and ZTD-weighted. Exporting a priori ZWD values, corrected for the interpolated station heights, is described in Section 5. Finally, Section 6 discusses the test and its results.

## 2. Multi-GNSS RTK Data Processing

With the plethora of new satellites and signals, additional observations are becoming available for high-precision positioning techniques. RTK positioning with instantaneous ambiguity resolution, as well as Precise Point Positioning (PPP) is the most common real-time precise positioning approaches. PPP requires a 20-min convergence time [26], whereas RTK delivers centimeter-level positioning services with a much shorter initialization time [27].

The DD equations with two stations, *A* and *B*, and two satellites, *r* and *s*, run as follows [28]:

(1) $$P_{AB,f}^{rs}=\rho_{AB}^{rs}+T_{AB}^{rs}+I_{AB,f}^{rs}+e_{AB}^{rs}$$

(2) $$\Phi_{AB,f}^{rs}=\rho_{AB}^{rs}+\lambda_{f}N_{AB,f}^{rs}+T_{AB}^{rs}-I_{AB,f}^{rs}+\epsilon_{AB}^{rs}$$

here ${\left(.\right)}_{AB}^{rs}={\left(.\right)}_{A}^{r}-{\left(.\right)}_{B}^{r}-{\left(.\right)}_{A}^{s}+{\left(.\right)}_{B}^{s}$ and *f* is the frequency band (*f* = 1, 2, ...). *P* and Φ are the pseudo-range code and phase observations, respectively (both in meters). ρ is the geometric distance between the satellites and receivers, *T* and *I* are the troposphere and ionosphere delays, respectively, all in meters. λ is the wavelength of the GNSS signal, *N* is the phase ambiguity, and *e* and ϵ are the unmodeled errors of the code and phase measurements, respectively.

By constructing DD measurement equations with a short baseline (<10 km), satellite ephemerides, ionosphere, and troposphere errors are effectively eliminated. However, the elimination of atmospheric delays is more challenging with medium-length baselines.

With even longer baselines, such as around 100 km or greater, the challenges become intractable. Over a very long baseline, the error terms in the DD equation, such as broadcast ephemeris errors, troposphere delays, and earth tides effects, are not negligible. In the observation, Equations (1) and (2), the error terms must be appropriately modeled. To exclude the ionosphere effect, dual-frequency measurements joined into a so-called ionospheric-free (IF) combination is used [29]. Therefore, in the following, by equation, we mean IF equations. However, one of the most significant error sources, the tropospheric delay, can be estimated with high precision using GNSS measurements as an unknown parameter. However, this increase in the number of unknowns would result in a longer initialization time.

## 3. Troposphere

Signal delays caused by the lowest layer of the Earth’s atmosphere are referred to as tropospheric delays. The troposphere can generate zenith pseudorange delays up to 2.5 m on the GNSS signals in the zenith direction. In contrast, for the satellites at low elevation angles, namely below 10 degrees, this delay can reach 20 m [13].

Instead of dealing with several independent line-of-sight delays for each satellite-receiver pair, all delays in the same area can be mapped to a single zenith total delay (ZTD) value, which, in turn, can be divided into hydrostatic and wet portions:

(3) $$\begin{array}{c} T=MF\cdot ZTD=\\ MF_{H}\left(el\right)\cdot ZHD+MF_{W}\left(el\right)\cdot ZWD+MF_{g}\left(el\right)\cdot \cot\left(el\right)\cdot \left(G_{N}\cdot \cos\left(\alpha \right)+G_{E}\cdot \sin\left(\alpha \right)\right) \end{array}$$

here el and α are the elevation and azimuth angle of a given satellite, respectively. MF is the mapping function, with $MF_{H}$ and $MF_{W}$ as the hydrostatic and wet mapping function, respectively. ZTD is the zenith total delay, which can be divided into zenith hydrostatic delay (ZHD) and zenith wet delay (ZWD). The gradients $G_{N}$ and $G_{E}$, along with the related gradient mapping function $MF_{g}$ account for the azimuthally inhomogeneous troposphere in the north-south and east-west directions, respectively [30,31].

The ZHD, dependent on the dry gas mixture ratio, is roughly 2.3 m. Whereas the zenith wet delay is on average 0.15 m in the global average. Even though the hydrostatic delay is greater than the wet delay, empirical models can estimate ZHD with a millimeter precision due to the reasonably constant gas mixture [13,32,33]. However, due to the lack of knowledge about the distribution of water vapor in the environment, the wet delay is far more difficult to model or eliminate.

Many zenith delay models have been proposed [4,13,34]. Different mapping functions based either on theoretical modeling or empirical data were proposed [32,35,36,37]; See Teunissen and Montenbruck for a comprehensive review of the troposphere models and mapping functions [38]. The Global Mapping Function (GMF) and Vienna Mapping Functions (VMF) are the two most common in GNSS data processing [39,40].

In this article, the hydrostatic and wet zenith delays are computed based on the Saastamoinen model using Equations (4) and (5) [13]. Moreover, for the mapping function, VMF3 was implemented [40].

(4) $$ZHD=0.0022768\frac{p}{1-0.00266\cos(2\phi )-0.00000028h}$$

(5) $$ZWD=0.0022768(\frac{1255}{T}+0.05)e$$

here *p* and *e* are the pressure and water vapor pressure in hectopascals, *T* is the temperature in degrees Kelvin, and ϕ and *h* are the station’s geographic latitude and ellipsoidal height, respectively. To achieve a priori zenith delays, global pressure and temperature models (GPT3) were introduced. This model tabulates annual and semi-annual mean values of atmospheric parameters required to predict ZTD components at a given place and time [40,41].

The above models can only partly compensate for the variability of wet delays [41,42]. Therefore, residual ZWD must still be treated as an unknown parameter in high-precision positioning. They can be considered practically constant for several minutes since it is rather steady in a limited region due to the relatively homogeneous water vapor content in the atmosphere [39,43,44]. For simplicity, the gradients are ignored as they are at the millimeter level [43,45]. Finally, to produce the best linear unbiased estimation (BLUE), the correct functional and stochastic model for the least squares processing should be defined, taking into account all of the unknowns as mentioned earlier [46].

## 4. Functional Model

Multi-GNSS positioning helps to increase the number of satellites, which is particularly beneficial in challenging environments. In the following, we consider a few advanced models for optimal integration of multi-GNSS observations with unknown parameters.

### 4.1. Tropospheric-Float Model

The design of the functional model should take into account that the equations mentioned in Section 2 are valid for the Code Division Multiple Access (CDMA)-based systems, i.e., GPS, Galileo, GLONASS M, BeiDou, and QZSS. Therefore, the functional model related to CDMA observations is as follows [38]:

(6) $$E\left[\begin{array}{c} P\\ \Phi \end{array}\right]=\left[\begin{array}{ccc} e⊗G & 0 & MF\\ e⊗G & \Lambda ⊗I_{m-1} & MF \end{array}\right]\left[\begin{array}{c} b\\ a\\ \delta ZWD \end{array}\right]$$

where *P* and Φ are the vectors containing DD-IF code and phase observables after correction for hydrostatic and wet zenith delays using Equations (4) and (5), *G* is the relative receiver-satellite geometry matrix, ⊗ denotes the Kronecker products, $e^{T}=\mathrm{ones}\;(1,f)$, Λ = diag$\left(\lambda_{1},...,\lambda_{f}\right)$ gives the diagonal matrix of wavelengths for *f* frequencies. *I* is the identity matrix of an order m−1 where *m* is the number of satellites, *b* is the baseline vector, *a* is a vector containing the DD integer phase ambiguities, and δZWD is the residual ZWD. MF contains mapping functions for projecting zenith wet delays to the satellite’s line-of-sight.

In contrast, the GLONASS system uses FDMA (Frequency Division Multiple Access) technologies, which means that each GLONASS satellite transmits on a different frequency. For more information about GLONASS, see [47]. As a result, fixing DD ambiguities can not be accomplished for the GLONASS in the same way as Equation (6). To address this problem, Equation (7) may be used to generate the functional model for GLONASS DD observations, which is quite similar to the DD CDMA model [48].

(7) $$E\left[\begin{array}{c} P\\ \Phi \end{array}\right]=\left[\begin{array}{ccc} e⊗G & 0 & MF\\ e⊗G & \Lambda ⊗L & MF \end{array}\right]\left[\begin{array}{c} b\\ a\\ \delta ZWD \end{array}\right]$$

in which *L* is a (m−1)×(m−1) full rank lower triangular matrix:

(8) $$L_{ii}=2848\frac{g_{i+1}}{a_{i+1}g_{i}}\;\;\;for\;\;i=1,...,m-1$$

(9) $$L_{ij}=2848\frac{\alpha_{j}a_{1(i+1)}}{a_{i+1}g_{j}}\;\;\;for\;\;i=j+1,...,m-1$$

The integers $\alpha_{i}$ and $\beta_{i}$ are given by $-\alpha_{i}a_{i+1}+\beta_{i}g_{i}=g_{i+1}$ in which $a_{i}=2848+f^{i}$, $f^{i}$ is the GLONASS satellite channel number, ∈[−7,+6], and $g_{1}=a_{1}$, $g_{i}=$ GCD$(a_{1},...,a_{i}\left(1<i\leq m\right)$.

When this model is combined with CDMA models, integer ambiguity estimation becomes possible. As a result, various types of multi-GNSS observations can be merged, and existing integer ambiguity resolution methods can be applied directly. Some AR techniques for medium and long-baseline RTK have also been published [22,49,50]. The most well-known of these is LAMBDA [51,52], which includes a reduction stage where the search space of ambiguities is reduced via decorrelation, followed by one of the `smart search’ algorithms to pick candidate ambiguity sets using the integer least-squares (ILS) criterion [53]. However, due to the presence of the matrix *L*, significant values of conditional variances of ambiguities for i=2m−2 and i=2m−3 for the FDMA model are produced [54,55]. As a result, Partial Ambiguity Resolution (PAR) should be used. It simply means that only a fraction of the ambiguities are resolved to integer values, while the remainder stays floating. There are a few conditions to decide whether ambiguity should be fixed. The criteria are, e.g., the variances of computed ambiguities, the duration of continuous valid data, and other considerations.

Furthermore, it is not easy to use this approach in its original form in long baseline RTK, where significant atmospheric residuals contribute to the RTK error budget. As previously noted, IF linear combinations (LC) are widely employed to remove the ionosphere’s effect. On the other hand, the ambiguous term cannot be separated into L1 and L2 terms. As a result, resolving carrier-phase ambiguities to an integer value is challenging without additional information. Another LC, the Melbourne−Wübbena (MW), which is usually employed to determine the ambiguities in this situation [56,57], cancels out the ionosphere term and geometry, including the tropospheric effect. It can restore redundancy and improve position determination through its correlation with the code and phase measurements. By averaging these LCs, we may estimate wide-line ambiguities. MW combination for CDMA and FDMA systems is as follows:

(10) $$L_{MW}=\phi_{WL}-P_{NL}=\frac{f_{1}\phi_{1}-f_{2}\phi_{2}}{f_{1}-f_{2}}-\frac{f_{1}P_{1}+f_{2}P_{2}}{f_{1}+f_{2}}=\lambda_{WL}N_{WL}$$

in which WL and NL refer to the Wide-Lane and Narrow-Lane combinations, respectively. Hence, $\lambda_{WL}=c/\left(f_{1}-f_{2}\right)$, indices 1 and 2 refer to the GNSS frequency bands and $N_{WL}=N_{1}-N_{2}$.

In this model, residual DD ZWD must be estimated alongside ambiguities and baselines. As a result, the mathematical model becomes less stable in this troposphere-float model. Including a priori knowledge of tropospheric delays in GNSS data as stochastic adjustments is one way to improve this model, which is called a troposphere-weighted model.

### 4.2. Tropospheric-Weighted Model

Assume that the tropospheric delay information on the rover station is available in a timely manner. This information can be used to fully compensate for troposphere delays for all satellites. Tropospheric delays can be calculated using long-term data when measurements are made within a permanent GNSS network. These estimations can then be interpolated to the position of a user station within the network, allowing for the correction of the user measurements. A large number of field tests in previous studies have well tested and demonstrated this network-RTK approach [25,58].

However, due to the long distances between the reference stations, interpolated and actual tropospheric delays might differ. As a result, if interpolated delays are not accurate enough, they may become a stumbling block for ambiguity fixing. The model can use a priori tropospheric delay as stochastic corrections in this situation. Consequently, residual tropospheric wet delay corrections are treated as pseudo-observations in the troposphere-weighted model, and their uncertainty is propagated in the stochastic model using a (co)variance matrix. A similar approach for ionospheric data was presented in an early essay by Bock et al. [59].

We include external residual tropospheric wet delay constraints as pseudo-observations to improve the model’s strength, using weighted least-squares. The amount of unknown troposphere parameters is as in the traditional RTK. If the a priori ZWD predicted via interpolation is accurate, the RTK performance with this approach should improve. This happens due to stochastic weighting, which allows for optimal use of a priori input information rather than considering them deterministic.

## 5. Stochastic Model

As previously stated, in addition to a suitable functional model, a realistic stochastic model for observables is required to achieve the best linear unbiased estimation of unknown parameters in the multi-GNSS data processing. Such a model would include various variances for each observation type, the correlation between different observables, and the satellite elevation dependence of the observables’ precision. The following is the stochastic model for an epoch:

(11) $$D\left[\begin{array}{c} P\\ \Phi \\ \delta ZWD \end{array}\right]=\left[\begin{array}{ccc} Q_{pp}⊗R & 0 & 0\\ 0 & Q_{\Phi \Phi }⊗R & 0\\ 0 & 0 & Q_{\delta ZWD}⊗R \end{array}\right]$$

in which $R=D^{T}W^{-1}D$ and $Q_{pp}=2\mathrm{diag}\left(\sigma_{p1}^{2},\sigma_{p2}^{2}\right)$, $Q_{\Phi \Phi }=2\mathrm{diag}\left(\sigma_{\Phi 1}^{2},\sigma_{\Phi 2}^{2}\right)$, $D^{T}=\left[\begin{array}{c} -e_{m-1},I_{m-1} \end{array}\right]$. As code and phase pseudo ranges and a priori tropospheric delay measurements are all in the units of length, matrix *D* is used to convert them to DD. Matrix *W* is the diagonal weight matrix, with its entries being based on the elevation of the satellites. The variances typically grow with decreasing elevation, and this helps to reduce the overall noise of the solution. The two parameters $Q_{pp}$ and $Q_{\Phi \Phi }$ are specific to the stochastic model for different systems. In most studies, unit weight variances are considered for different types of equations. To define realistic (co)variance components for each system, the Least-Squares Variance Component Estimation (LS-VCE) method can be used [20,60,61]. If one considers the covariance matrix as a linear combination of *q* (co)variance components, then:

(12) $$D\left(y\right)=Q_{yy}=Q_{00}+\sum_{k=1}^{q}\sigma_{k}Q_{k}$$

where $Q_{00}$ is the known part of the variance matrix, $\sigma_{k}$ is an unknown (co)variance components, and $Q_{k}$ is known symmetric and positive definite cofactor matrices. The realistic (co)variance matrix of observations can be constructed using the iterative Equation (13).

(13) $$\hat{\sigma }=N^{-1}l$$

in which:

(14) $$n_{ij}=\frac{1}{2}tr\left(Q_{i}Q_{yy}^{-1}P_{A}^{⊥}Q_{j}Q_{yy}^{-1}P_{A}^{⊥}\right)$$

are the elements of matrix *N*, and the components of vector *l* are defined as follows:

(15) $$l_{i}=\frac{1}{2}{\hat{e}}^{T}Q_{yy}^{-1}Q_{i}Q_{yy}^{-1}\hat{e}$$

where $\hat{e}$ is the least-squares estimator of residuals, and $P_{A}^{⊥}$ is a projector that projects onto the range space of $A^{⊥}$. The iterative process in Equation (13) continues until the difference between two consecutive solutions is less than the tolerance ϵ. Using the estimated (co)variance matrix in the least-squares adjustment allows one to obtain a realistic precision of the unknowns. Due to the large size of the A matrix, as in [61], the multivariate linear model was used to reduce the computational load and memory usage for VCE. In other words, the model is suggested for a number of successive epochs in each group. The final calculation of the variances will then be based on the mean value of the estimated variances.

We previously noted that we treat δZWD as pseudo-observations. The variances of all pseudo-observations is assumed to be the same, i.e., $\sigma_{ZWD}^{2}$. Other possible effects, such as a dependency on the elevation angle or time correlation, have not been considered. The a priori tropospheric information is considered to be wholly known if $\sigma_{ZWD}=0$ (or infinite weight) so that the solution becomes entirely equivalent to that of the troposphere-fixed model. On the other hand, if $\sigma_{ZWD}=\infty$ (or zero weight), the a priori tropospheric information does not affect the model’s solution (tropospheric errors are assumed to be completely unknown), and the result is the same as the troposphere-float model. When a troposphere-weighted model is employed, the tropospheric adjustments have more space for uncertainty, allowing for correct ambiguity resolution. For estimating the $\sigma_{ZWD}$ value, the RMSE between the estimated and interpolated values for the permanent stations of the network around the rover station can be used.

## 6. Modified ZTD Interpolation for Height Differences

The DD tropospheric delays are estimated for each baseline in an RTK Network. Each DD tropospheric delay refers to a pair of stations and a pair of satellites, with mapping functions used to convert each station’s ZTD to STD. Then, these DD slant tropospheric delays will be interpolated between the nearest reference stations to the rover position. For the purposes of such interpolation, the same pair of satellites should be used for all baselines included in the calculation. Hence, it is preferable to interpolate the ZTD for the stations in the given RTK network.

Since the ZTD value is a direct function of the station’s height, it is expected that the interpolation accuracy of ZTD will get worse for pairs of stations with large differences in altitude. It is also well known that the hydrostatic component directly depends upon the height, accounting for roughly 90% of the total tropospheric delay.

In [62], a technique that reduces the effect of station height on tropospheric delay interpolation is proposed. In accordance with this method, the discrepancy between the altitudes of reference and rover stations is neglected. All the involved stations are treated as if being at the same level as the rover; to this end, all the tropospheric delays are “recomputed” to this level. To accomplish this, the reference stations’ altitude and latitude are imported into the Saastamoinen Equation (4). At each epoch, the dry part of the tropospheric delay is subtracted from the total tropospheric delay. The wet part of the tropospheric delay will remain. Then, using the Saastamoinen equation with the latitude of surrounding reference stations and the height of the rover station, we get the dry delays as if the reference stations were situated at the same height as the rover station. These results will be added to the wet delays estimated in the previous step to obtain the total tropospheric delays under new conditions. The following is a simplified formula:

(16) $$\begin{array}{c} ZTD_{i}^{\prime }=ZTD_{i}-0.0022768\frac{P_{i}}{1-0.00266\cos\left(2\phi_{i}\right)-0.00000028h_{i}}\\ +0.0022768\frac{P_{i}}{1-0.00266\cos\left(2\phi_{i}\right)-0.00000028h_{r}} \end{array}$$

where $ZTD_{i}^{\prime }$ and $ZTD_{i}$ are the corrected and estimated ZTD for the *i*th reference station, respectively, $\phi_{i}$ is the latitude of the *i*th reference station, $h_{i}$ is the reference station’s height, $h_{r}$ is the rover station height, and $P_{i}$ is the pressure computed for the *i*th reference station using GPT3 model.

Interpolation can be performed after the revised ZTD values have been obtained. The Linear Combination Model, the Distance-Based Linear Interpolation Method, and Kriging are some of the methods for interpolating ZTDs [27,63].

Ordinary Kriging, one of the most common Kriging interpolation methods, was used in this study. It implies that the attribute has a constant mean across the entire spatial domain, which is reasonable for the wet delay in a local area, but the mean value is unknown. It is also assumed that the unknown point’s predictor is a weighted linear function of all the data. Ordinary Kriging’s covariance can be defined by a single covariance function valid for the entire spatial domain. The covariance function accounts for the variation of observed data because the values at two neighboring sites are highly correlated, whereas values at two positions far apart are not. If the tropospheric ZWD estimations at the stations are used as the Kriging interpolation’s known values $z_{i}$, and $w_{i}$ is the ordinary Kriging weight of the *i*th station’s ZWD, the purpose is to determine the unknown variable $z_{0}$ (ZWD at user stations), as in Equation (17):

(17) $$\begin{array}{c} z_{i}=\sum_{i}w_{i}z_{0} \end{array}$$

The following linear equations can be used to compute the weight, using $h_{ji}$ as the distance between the known points, $h_{j0}$ is the distance between a known point *j* and an unknown point, $C(h_{ji})$ is the data-to-data covariance function, and $C(h_{j0})$ is the data-to-estimation point covariance function.

(18) $$\begin{array}{c} \Sigma_{i}w_{i}-C\left(h_{ji}\right)-C\left(h_{jo}\right)=0\\ \Sigma_{i}w_{i}=1 \end{array}$$

The following equation can be used to derive a variogram function $\gamma_{ji}$ from the covariance function:

(19) $$\begin{array}{c} \gamma_{ji}=C\left(0\right)-C\left(h_{ji}\right) \end{array}$$

The weight $w_{i}$ is known to be dependent on the spatial fluctuations of all measured points as well as the distances between the measured points and predicted location. As a result, the spatial correlations must be measured using a covariance function on the network’s stations. The theoretical variogram model is a function that describes spatial relationships between the measured values and the projected location. The variogram model in this study is a Gaussian model [64]:

(20) $$\begin{array}{c} C\left(h_{ji}\right)=bexp\left(-\frac{h_{ji}^{2}}{a^{2}}\right) \end{array}$$

Initial values in the ideal solution are sensitive to the variogram fitting procedure. The variogram fitting procedure will begin with automatic initial values *b* = max(γ) and *a* = max(h)∗2/3), if initial values are unknown. The Newton–Raphson method is utilized to obtain the optimum values of these parameters with least squares [65]. It should be noted that the distances between two points, *i* and *j*, are calculated as Euclidean distances since the stations are practically on a flat surface. Because this is a regional area, the spheroidal distances are ignored for simplification. The unknown variable can be obtained via Equation (17) after the weights $w_{i}$ have been determined, and its variance is provided by:

(21) $$\begin{array}{c} var\left(z_{0}\right)=C\left(0\right)\sum_{i}w_{i}C\left(h_{0i}\right) \end{array}$$

As previously stated, the distance-based Kriging method is the most well-known interpolation method. However, it is dependent on the semivariogram. As a result, when the covariance function is not known, the Low-Order Surface Model (LSM), which is more straightforward, can be used [66]. This method is characterized by fitting a low-level surface to the datasets. The corrected ZTDs of at least four stations specified with the *i* index can be used to interpolate the ZTD values of the rover station using Equation (22), where *X*, *Y*, and *Z* are the station’s coordinates.

(22) $$ZTD_{i}=X_{i}\alpha +Y_{i}\beta +Z_{i}\gamma +\delta$$

The least squares can evaluate the fitted surface coefficients, and then by replacing the coordinates of the rover station, the interpolated ZTD for the user can be achieved.

## 7. Results and Analysis

**Figure 1:**
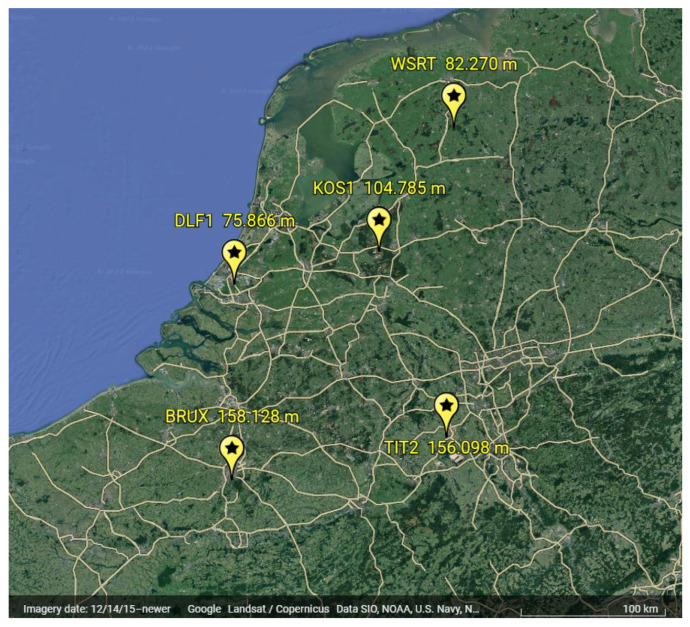
Position of the stations with their heights.

To find out how this method works, rinex daily observations for five IGS stations that correspond to 3−9 January 2021 were chosen as an example. The geographical locations and altitude of these stations can be seen in Figure 1. Four of these stations (DLF1, WSRT, BRUX, and TIT2) were taken as reference stations, while KOS1 was treated as the rover station. In total, six baselines between reference stations as well as a baseline DLF1-KOS1 are involved in this analysis. These baselines are between 100 and 200 km. The purpose of selecting one of the IGS stations as a rover station was to compare the results and efficiency of the suggested methods by comparing our results to the known ZTD values and precise positions of IGS stations. The Rinex files contained measurements of four constellations: GPS, Galileo, GLONASS, and BeiDou satellites. The selected options of our processing strategy are summarized in Table 1.

**Figure 2:**
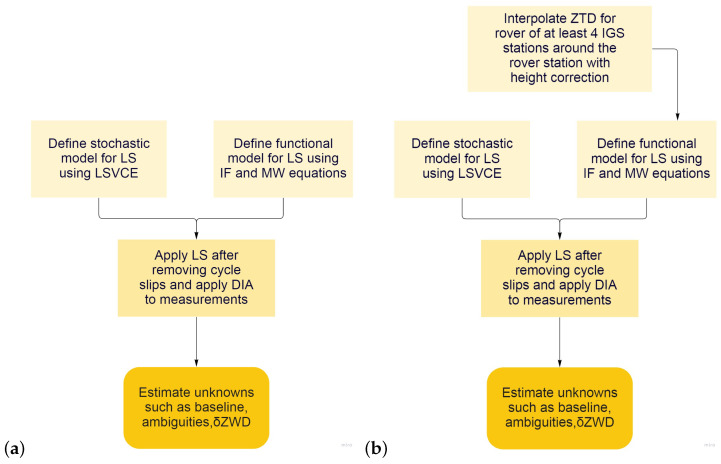
Simple flowchart for the estimation producer: (**a**) Tropospheric-float model; (**b**) Tropospheric-weighted model.

The actions we have taken to prepare and process raw data and to estimate ZTD values using the least squares approach are depicted in a schematic representation below (Figure 2).

Even though this research is performed in post-processing mode, its strategies are fully customized for real-time applications. However, the construction of functional and stochastic models covering all possible signal options available with four constellations still needs to consider a few more issues as follows:

First is the cycle slips, which happens when the receiver tracking loops lose the lock of the signal. Cycle slips enter the processing as uncompensated jumps of phase measurements on one or more signals. To achieve cm-level accuracy, such undesirable events must be detected and handled before the carrier phase observations are used. Although multiple algorithms have been proposed to detect and repair cycle slips [67], error-free functioning and high sensitivity of such algorithms remain a challenge. In particular, the wide-lane cycle slip detection method was proposed [67,68]. A long-wavelength (such as 86.2 cm for GPS L1-L2 wide-lane) allows direct cycle slip detection at any epoch when proper code measurements are available. However, at low satellite elevation, the pseudorange noise may be significantly higher than in normal conditions due to multipath or ionospheric disturbances. In such circumstances, small cycle slips such as one or two cycles could pass undetected. To address this challenge, we used an additional cycle slip detection method [68]. In accordance with this method, the geometry free (GF) equation and ionospheric-free observation corrected for the computed geometrical distance (IF-OMC Ionospheric free Observed Minus Calculated) should be calculated for DD phase observations. The simultaneous use of these two formulas compensates for each other’s shortcomings [68].

After this step, DIA is done. Teunissen developed the DIA method for detecting, identifying, and adapting incorrectly modeled errors in GNSS data processing, and it has been used in a variety of GNSS applications [28]. A real-time DIA technique is used in this contribution to detect outliers, particularly those induced by tropospheric delays. After the problems are identified, those observations are removed.

After removing cycle slips and blunders, the functional and stochastic models for multi-GNSS processing could be defined. In this way, the results will be more accurate. The variances of different observations was calculated using the LS-VCE method to achieve the BLUE of unknowns. The data of these baselines are divided into 288 groups (ten consecutive epochs in each group) for each day’s implementation of LS-VCE, which increases the number of estimates to be sufficient for more reliable results. This is done to properly weigh the observations and come up with a realistic stochastic model. However, a group may be empty due to primary criteria such as a low elevation angle, outlier identification, or even the absence of satellites. For some days, there might be fewer computed (co)variances. Finally, the observation variances are provided as the mean value of these calculated variances for each group of 10 successive epochs. The weekly set of Rinex files of these stations were processed to ensure these values. The mean results are provided in Table 2. Using these estimated variances in the stochastic model allows us to derive a realistic stochastic description of the unknowns.

**Figure 3:**
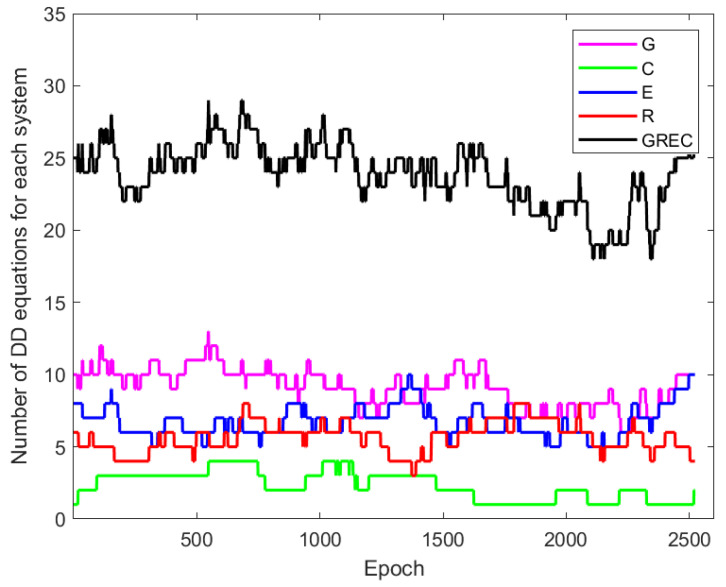
Number of DD equations for each system for baseline BRUX-DLF1 for 3-Jan-2021 (G: GPS, R: GLONASS, E: Galileo, C: BeiDou).

The epoch-wise least-squares adjustment for whole days was applied after defining the functional and stochastic models based on the equations discussed previously. It should be noted that the reference satellite for double differencing was chosen separately for each constellation. The number of DD equations that were made for each system in baseline BRUX-DLF1 is shown in Figure 3. It is easy to see how much multi-GNSS might improve redundancy.

Furthermore, LAMBDA’s partial ambiguity resolution method was employed to fix the float ambiguities to integer numbers, keeping the less precise GLONASS observations as the float. The remaining unknown parameters, such as position components and tropospheric delay, can be accurately estimated once the phase ambiguities are resolved to integer values. The δZWD and position in float and fixed solutions were computed for the rover station.

The IGS ZTD files are used to evaluate the estimated tropospheric delays for all the baselines. When comparing the ZTD results obtained from the troposphere-float model treating δZWD as an unknown to the IGS ZTD products, the mean RMSE for all the baselines was about 11.95 mm. At the same time, it was equal to 14.02 mL without considering the δZWD. It means that the computed tropospheric delay matches the IGS results more closely when δZWD compensates for biases in the Saastamoinen modeling. Table 3 shows the findings for both situations over a period of seven days.

**Figure 4:**
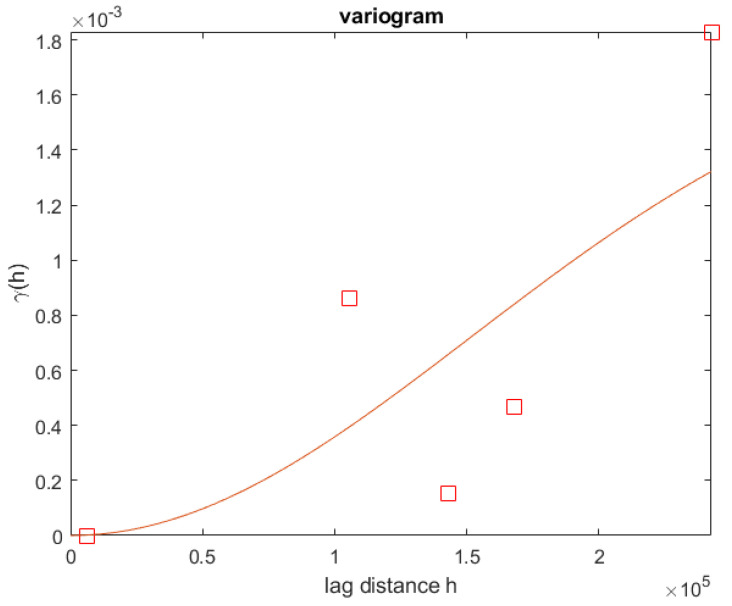
Sample variogram for the network (squares symbols) with the fitted model (solid curve).

**Figure 5:**
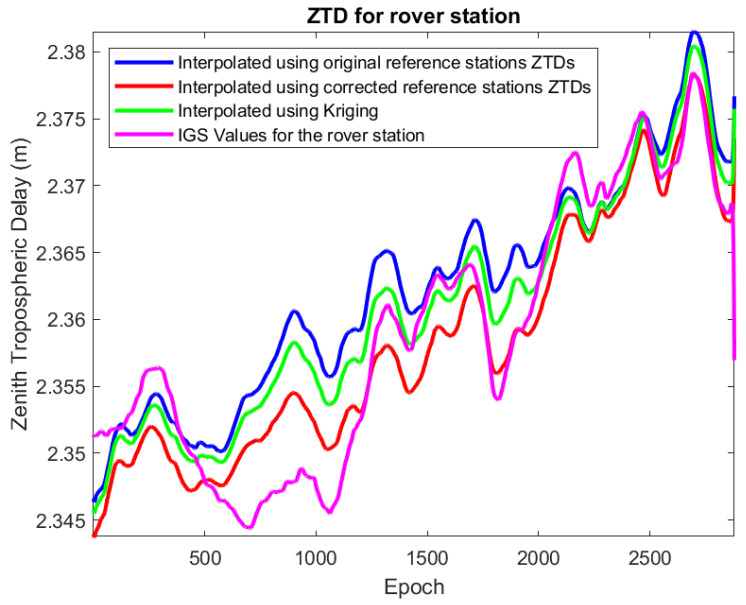
IGS Zenith tropospheric delays for rover station compared to the interpolated values.

In the next phase, for the tropospheric-weighted model, the ZTD of the four reference stations was interpolated using Equation (22) to obtain the interpolated ZTD for the rover station. This technique was carried out on a daily basis for this week. Because the results for all days were somewhat similar, only the first day’s result is reported. The RMSE value compared to the IGS ZTD for the rover station was estimated to be equal to 5.4 mm. Then the ZTDs of the reference stations were corrected for the height differences using the method explained in Section 6 and Equation (16). The LSM interpolation of these corrected ZTD has an RMSE of 3.4 mm. To evaluate the performance of the Kriging interpolation, data from the mentioned week for these four stations were interpolated [69]. The sample variogram was computed and presented in Figure 4. Kriging interpolation results in a 4.3 mm RMSE. Based on these findings, it can be concluded that the LSM approach produced better interpolation results when the height-corrected ZTD was used. Figure 5 shows a comparison of the interpolated ZTD for the rover station.

**Figure 6:**
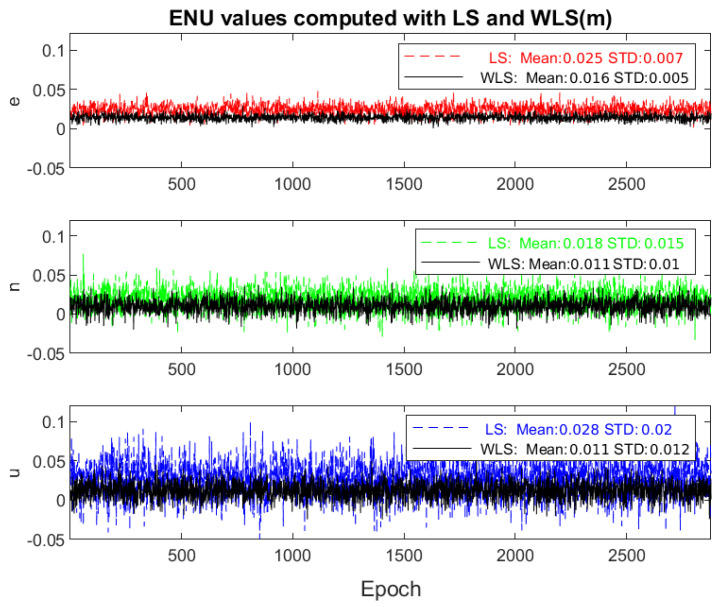
NEU values for the rover station for fixed least-squares and weighted least-squares solutions with their means and standard deviations for 3−9 January 2021.

We mentioned that the interpolated tropospheric delays could be added to the model to improve it. The ZTD of the reference station DLF1 and the corrected interpolated ZTD for the rover station are entered as stochastic corrections to the tropospheric delays in the WLS processing. The difference between the IGS and interpolated ZTDs can be treated as the standard deviation for these a priori data ($\sigma_{ZWD}=3.4$ mm) in defining the stochastic model. Figure 6 shows the rover’s mean position error for a week after fixing the ambiguities in an epoch-wise method with LS and WLS. As the mean and standard deviations of the coordinates show, adding a priori information of the residual ZWD improves the rover station’s position in the case of a long baseline.

Furthermore, the success rate can be another criterion for evaluating the LS and WLS performance. Table 4 shows the average success rate for integer ambiguity resolution in both cases for each day. While the LS average success rate for integer ambiguity resolution is 0.976, the WLS success rate is 0.999.

**Figure 7:**
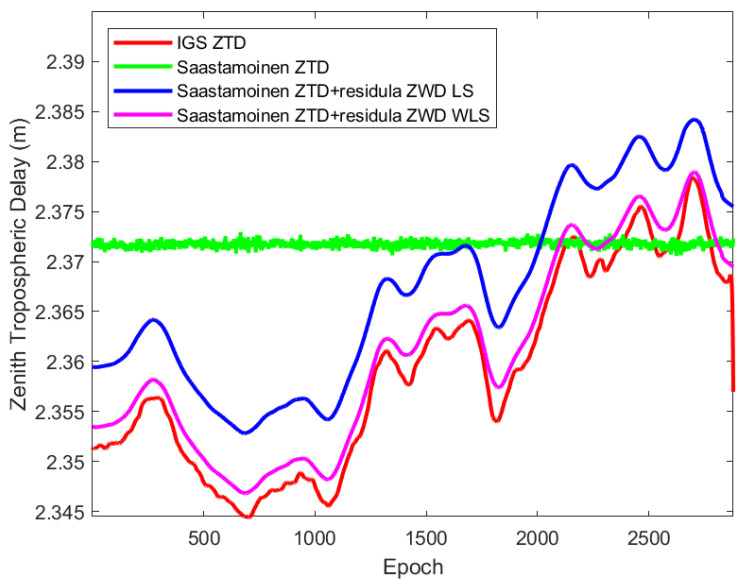
Zenith Tropospheric Delay from IGS and computed with LS and WLS.

In Figure 7, the ZTD for the rover station from IGS files is compared to the sum of the zenith hydrostatic, wet, and residual wet delays computed from LS and WLS methods, as well as the sum of ZHD and ZWD, calculated with the Saastamoinen model. As can be seen, the WLS-calculated ZTD is comparable to the IGS values; their RMSE value amounts to 2 mm, while the RMSE for LS-calculated ZTDs goes up to 8 mm.

## 8. Conclusions

When multi-constellation GNSS is utilized, the number of visible satellites increases. Degrees of freedom and geometry strength can be enhanced by processing their observation equations. Hence, it is no surprise that Multi-GNSS positioning services are now being used worldwide. Using proper modeling for these code and phase observations, the ionosphere’s impact can be largely minimized. The troposphere, on the other hand, affects GNSS signals. The troposphere structure should be modeled to improve positional accuracy. The DD tropospheric delay is calculated by combining DD measurements from multiple positioning systems such as GPS, GLONASS, Galileo, and Beidou and estimating the unknowns using the least-squares approach. Thus the estimated ZTDs compare well to the tabulated IGS ZTD values. These findings suggest that the computed ZTD values are reliable. Hence, they can be employed in the weighted least-squares approach, meteorology prediction, and tropospheric tomography as a priori known troposphere zenith delays.

To acquire a valid ZTD for the rover station, the interpolation of ZTD from nearby IGS stations corrected for height with the LSM method can be used. This approach does not presuppose a covariance function such as the Kriging interpolation technique. Moreover, this method is less computationally intensive than NRTK. Furthermore, ZTD information for IGS stations can be obtained reasonably through IGS products or near real-time services, such as E-GVAP (http://egvap.dmi.dk (accessed on 20 April 2022)), and it does not require specialized software. A priori ZTD of both reference and rover stations would strengthen the model in WLS. Thus the estimated rover position is up to 50% more accurate compared to the least-squares with the troposphere float model. It also has a better success rate for the IAR, close to one, owing to the closeness of the interpolated ZTDs to tabulated IGS ZTD values.

## Figures and Tables

**Table 1:** Summary of the strategy of data processing.

Item	Strategy/Value
Positioning mode	Static/Kinematic
Constellation Frequency	GPS ($L_{1}\&L_{2}$)
	GLONASS ($G_{1}\&G_{2}$)
	Galileo ($E_{1}\&E_{5}$)
	BeiDou ($B_{1}\&B_{2}$)
Satellite orbits/clocks	IGS(Code)
Combination	Ionosphere free
Observation	Double difference
Unknowns	δX,δY,δZ,δZWD,N
Ambiguity Resolution	Fixed (Partial resolution with the success rate criterion 0.995)
Elevation cutoff angle	$10^{\circ }$
Interval	30 s
Weighting Strategy	Elevation-dependent

**Table 2:** Estimated STD of phase and code observations (meter).

System	${\hat{\sigma }}_{P}$	${\hat{\sigma }}_{\phi }$	${\hat{\sigma }}_{\mathrm{MW}}$
GPS	0.41	0.0017	0.08
GLONASS	0.70	0.0024	0.15
Galileo	0.26	0.0017	0.06
BeiDou	0.37	0.0011	0.09

**Table 3:** The RMSE between IGS ZTD and: (**a**) Estimated ZTD with δZWD and (**b**) Estimated ZTD without δZWD.

(a)								
	**Day**	1	2	3	4	5	6	7
**Baseline**								
1		12.86	12.82	12.88	12.70	12.81	12.84	12.91
2		12.67	12.64	12.62	12.66	12.54	12.60	12.64
3		11.76	11.67	11.62	11.70	11.60	11.65	11.76
4		13.39	13.26	13.30	13.39	13.29	13.47	13.27
5		10.57	10.52	10.41	10.46	10.48	10.38	10.53
6		10.79	10.70	10.74	10.80	10.74	10.91	10.73
**Mean**		12.00	11.935	11.93	11.95	11.91	11.975	11.97
**Total Mean**		11.95						
(**b**)								
	**Day**	1	2	3	4	5	6	7
**Baseline**								
1		14.93	14.98	15.00	14.91	14.98	14.92	14.99
2		13.38	13.39	13.35	13.35	13.29	13.35	13.37
3		12.96	12.95	12.96	12.92	12.84	12.88	12.97
4		14.96	14.97	15.01	14.98	14.89	14.99	14.97
5		15.43	15.41	15.46	15.45	15.39	15.38	15.41
6		12.54	12.48	12.51	12.56	12.51	12.55	12.56
**Mean**		14.03	14.03	14.05	14.03	13.98	14.01	14.04
**Total Mean**		14.02						

**Table 4:** Integer ambiguity resolution success rate.

Method	Day							Mean
	1	2	3	4	5	6	7	
**LS**	0.996	0.990	0.986	0.981	0.989	0.991	0.984	0.988
**WLS**	0.999	1	0.998	0.999	1	1	0.998	0.999
